# Predictive value of pan-immune-inflammation value and ultrasonographic parameters in cervical lymphadenopathy: development of a novel clinical scoring system

**DOI:** 10.1007/s00405-026-10282-0

**Published:** 2026-05-05

**Authors:** Mehmet Aslan, Baransel Algan

**Affiliations:** 1 Department of Otorhinolaryngology Head and Neck Surgery, Inonu University Faculty of Medicine, Malatya, Turkey; 2 Faculty of Medicine, Department of Otorhinolaryngology Head and Neck Surgery, Inonu University, Malatya, 44000 Turkey

**Keywords:** Cervical lymphadenopathy, Pan-immune-inflammation value (PIV), Platelet-to-lymphocyte ratio (PLR), Cervical ultrasound

## Abstract

**Objective:**

Accurate preoperative differentiation of cervical lymphadenopathy (LAP) is essential for optimal surgical planning. This study aimed to evaluate the contribution of preoperative pan-immune-inflammation value (PIV), platelet-to-lymphocyte ratio (PLR), demographic characteristics, and ultrasonographic findings in predicting malignant histopathology in patients undergoing excisional biopsy for cervical LAP, and to develop a practical clinical risk score.

**Materials and methods:**

This retrospective cohort study was conducted at a tertiary referral center between January 2014 and December 2025. Patients who underwent excisional cervical lymph node biopsy with available preoperative ultrasonography and complete blood count data were included. The primary outcome was malignant versus non-malignant (reactive or granulomatous) histopathology. Candidate predictors included age, sex, neck level, ultrasonographic features (hilum loss, conglomeration, roundness index, and longest diameter), and inflammatory indices (PIV and PLR). Independent predictors were identified using multivariable logistic regression analysis, while discriminatory performance and optimal cut-off values were assessed using receiver operating characteristic (ROC) analyses.

**Results:**

Of the 481 patients included, 184 (38.2%) had reactive, 91 (18.9%) granulomatous, and 206 (42.8%) malignant histopathology. Patients with malignancy were older (median age, 51.5 years) and more frequently male (65.5%) (both *p* < 0.001). Multivariable analysis demonstrated that conglomeration (OR, 6.18), male sex (OR, 3.51), Level V localization (OR, 7.77), increased roundness index (OR, 2.33), age (OR per year, 1.04), and PIV (OR per unit, 1.001) were independently associated with malignancy. Although PIV showed moderate discriminatory ability (AUC, 0.624), it achieved high specificity (83.3%) at a cut-off value > 567.1. A six-variable, points-based clinical scoring system (range, 0–11) effectively stratified malignancy risk (7.4% in the low-risk group and 83.1% in the high-risk group).

**Conclusion:**

Malignancy risk in cervical lymphadenopathy can be reliably predicted through the combined assessment of demographic characteristics, ultrasonographic patterns, and the Pan-Immune-Inflammation Value. Conglomeration, Level V localization, advanced age, and elevated PIV emerged as the strongest independent predictors of malignancy. However, multicenter external validation is required before the proposed risk score can be implemented in routine clinical practice.

## Introduction

The differential diagnosis of cervical lymphadenopathy remains a persistent challenge in otolaryngology. While distinguishing between reactive lymphadenitis, granulomatous diseases, and malignancy is essential for surgical planning and path of treatment, current imaging modalities like ultrasonography often lack the specificity and definity that required to rule out malignancy certainly.

Systemic inflammation is deeply linked to tumor progression. Consequently, biomarkers derived from routine complete blood counts, such as the platelet-to-lymphocyte ratio (PLR) and the pan-immune-inflammation value (PIV), have emerged as prognostic indicators in head and neck cancers [1–5]. Increased PIV values have been shown to be associated with poor prognosis in patients with breast cancer, laryngeal and pharyngeal tumors, nasopharyngeal carcinoma, and head and neck squamous cell carcinoma [6–9]. The findings of two studies indicated that the overall survival rate was diminished when the cut-off value of PIV was established at 404 in a study conducted on patients diagnosed with head and neck squamous cell carcinoma [9], and at 512 in a study conducted on patients diagnosed with nasopharyngeal carcinoma [8]. A study was conducted on patients with HPV-negative head and neck carcinoma, with the objective of determining the statistical significance of PLR in detecting occult metastasis. The study found that when the accepted cut-off value of 116.1 was applied, PLR exhibited statistical significance in this regard [10]. These markers are cost-effective and readily available; however, their utility is largely restricted to prognostic modeling rather than preoperative diagnosis.

Data integrating these systemic inflammatory markers with sonographic findings to predict histopathology in neck masses remain limited. Researches mainly focuses on outcomes rather than the diagnostic differentiation of the mass itself. Although PLR and PIV have been investigated primarily as prognostic markers in various malignancies, their diagnostic role in distinguishing benign from malignant cervical lymphadenopathy has not been previously reported.

This study investigates the diagnostic worth of preoperative demographic, sonographic, and hematologic parameters—specifically PLR and PIV—in a cohort undergoing excisional cervical lymph node biopsy that is done by tertiary referral center. We aimed to develop a multiparametric predictive model to improve preoperative risk assessment and optimize surgical decision-making or clinical follow-up.

## Materials and methods

### Study design and ethical approval

This retrospective cohort study was conducted at a tertiary university hospital. Ethical approval was obtained from the XXX University Ethics Committee (Approval No: 2025/8738). The study was conducted in accordance with the principles of the Declaration of Helsinki. Due to the retrospective nature of this study, the requirement for informed consent has been waived.

### Study population

Patients who underwent excisional biopsy for cervical lymphadenopathy at our institution between January 2014 and December 2025 were screened for inclusion.

Inclusion criteria were:

1. Complete preoperative ultrasonography (USG) and hematological data (complete blood count) available,
2. Final histopathology result available.

Exclusion criteria included:

1. Missing preoperative data,
2. Known hematologic malignancies (e.g., leukemia, lymphoma) prior to biopsy,
3. Previous neck surgery or radiotherapy, which could alter lymph node structure and hematologic parameters.

### Data collection

All data were extracted from electronic medical records and hospital information systems. The following variables were included in the analysis:

## Dependent variable (outcome)

- The histopathological diagnosis was categorised into three groups:
  1. Reactive lymphadenitis.
  2. Granulomatous lymphadenitis (e.g., tuberculosis, sarcoidosis).
  3. Malignancies (e.g., metastatic carcinoma, lymphoma).

## Independent Variables (Predictors)

- **Demographics**: Age, sex (male/female).
- **Clinical**: Lymph node location (neck levels I–VII).
- **Ultrasonography**:

-   ○ Hilus echogenicity (faded, pronounced).
-   ○ Conglomeration (absent, present).
-   ○ Roundness index (normal, increased).
-   ○ Longest axial diameter (mm).

- ** Hematological markers**

-   ○ Platelet-to-lymphocyte ratio (PLR).
-   ○ Pan-immune-inflammation value (PIV) calculated as (Neutrophil × Platelet × Monocyte) / Lymphocyte.

## Sample size and power calculation

The sample size was determined based on the ‘Events Per Variable’ (EPV) rule for multivariable logistic regression analysis. To ensure the stability and reliability of the regression model, a minimum of 10 events (malignant cases) per independent variable is traditionally recommended. In our study, 206 malignant cases were analyzed against 9 independent variables, resulting in an EPV ratio of 22.8. This ratio significantly exceeds the minimum threshold, providing sufficient statistical power to minimize the risk of overfitting and to ensure the validity of the predictive model.

### Statistical analysis

All analyses were performed using IBM SPSS Statistics (version 26, IBM Corp., Armonk, NY, USA).

#### Descriptive Statistics and Normality

The Kolmogorov–Smirnov test was employed to ascertain the normality of the continuous data. Given the skewed distribution of the continuous variables, the median with interquartile range (IQR: 25th–75th percentiles) was employed for all variables.

#### Group Comparisons

For the comparison of continuous variables among the three histopathological groups (reactive, granulomatous, and malignant), the Kruskal–Wallis test was used. When statistical significance was observed, post-hoc pairwise comparisons were conducted with Bonferroni correction to avoid Type I error. The Chi-square test was used to compare categorical variables, and where necessary, Bonferroni-adjusted pairwise comparisons were performed.

#### Predictive Modeling (Logistic Regression)

A binary logistic regression analysis was conducted for the purpose of identifying independent predictors of malignancy. The outcome variable was classified as either malignant or non-malignant, with the latter category encompassing reactive and granulomatous combined manifestations. First, a univariate analysis was performed; variables with a p-value < 0.10 were then included in the multivariable binary logistic regression model. Results were reported as Odds Ratios (ORs) with 95% confidence intervals (CIs). The model’s fit was evaluated using the Hosmer-Lemeshow goodness-of-fit test and Nagelkerke pseudo-R².

#### Diagnostic Performance and ROC Analysis

Receiver Operating Characteristic (ROC) curve analysis was performed to evaluate the diagnostic ability of continuous predictors (age, PIV, and longest diameter) in predicting malignancy. The area under the curve (AUC) was calculated. The optimal cut-off values were determined by maximising the Youden Index (Sensitivity + Specificity − 1). For both the derived cut-offs and categorical predictors (e.g., conglomeration, anatomical level), diagnostic performance was evaluated by calculating sensitivity, specificity, positive predictive value (PPV), and negative predictive value (NPV). To develop the clinical scoring system, weighted points were assigned to each independent predictor based on the relative magnitude of their regression coefficients (β) obtained from the multivariable logistic regression model. The coefficients were rounded to the nearest integer to facilitate practical clinical application.

#### Significance Level

A two-sided p-value of < 0.05 was considered statistically significant for all analyses.

### Ethics and reporting

The study design, data collection, and analysis were carried out in accordance with the ethical guidelines for human research. The reporting follows the STROBE guidelines for observational studies.

## Results

### Demographic and clinical characteristics

A total of 481 patients who underwent cervical lymph node excision were included in the study. Based on histopathological evaluation, patients were divided into three groups: reactive lymph node (*n* = 184, 38.2%), granulomatous lymphadenitis (*n* = 91, 18.9%), and malignancy (*n* = 206, 42.8%). The median age of patients in the malignant group [51.5 years (IQR: 34.2–65.0)] was statistically significantly higher than that of the reactive [33.5 years (IQR: 19.8–48.0)] and granulomatous [30.0 years (IQR: 19.5–49.0)] groups (*p* < 0.001). When gender distribution was examined, male predominance (65.5%) was observed in the malignant group, while female predominance (70.3%) was observed in the granulomatous group (*p* < 0.001). The most common specific diagnoses in the malignancy group were Hodgkin’s Lymphoma (*n* = 100) and B-cell Lymphoma (*n* = 60). The median age of B-cell lymphoma patients [62.0 years (IQR: 52.8–71.3)] was significantly higher than that of Hodgkin lymphoma patients [35.5 years (IQR: 20.8–53.3)] (*p* < 0.001).

### Comparison of radiological and laboratory findings

The comparative analysis of ultrasonographic (USG) and laboratory parameters between groups is presented in Table 1. The median longest diameter of malignant lymph nodes [26.0 mm (Interquartile Range, IQR: 18.0–38.0)] was significantly higher than that of benign [22.0 mm (IQR: 17.0–29.0)] and granulomatous [26.0 mm (IQR: 20.0–33.0)] lymph nodes (*p* = 0.001). With regard to USG morphological features, the loss of fatty hilum, conglomeration, and an increased roundness index was found to be statistically significantly more prevalent in the malignant group than in the other groups (*p* < 0.001 for all parameters). Conglomeration was observed in 65.5% of malignant cases, while it was only seen in 19.6% of benign cases.

**Table 1:** Comparison of demographic, ultrasonographic, and laboratory characteristics of patient groups. Statistical significance (*p* < 0.05). a, b, c: Groups with different letters in the same row have statistically significant differences (Post-hoc Bonferroni corrected tests, *p* < 0.05). Groups with the same letter have no difference

Variables	Reactive (*n* = 184)	Granulomatous (*n* = 91)	Malignant (*n* = 206)	*p* Value
**Age (Year)**, Median (IQR)	33.5(19.8–48.0)^a^	30.0(19.5–49.0)^a^	51.5(34.2–65.0)^b^	< 0.001*
**Gender**, *n (%)*				< 0.001*
Male	92 (%50.0)^a^	27 (%29.7)^b^	135 (%65.5)^c^	
Female	92 (%50.0)^a^	64 (%70.3)^b^	71 (%34.5)^c^	
**USG Findings**				
Longest Diameter (mm), Median (IQR)	22.0(17.0–30.0)^a^	26.0(21.0–33.0)^b^	26.0(18.0–36.0)^b^	0.001*
Hilus Fading	44 (%23.9)^a^	29 (%31.9)^a, b^	91 (%44.2)^b^	< 0.001*
Conglomeration	36 (%19.6)^a^	28 (%30.8)^a^	135 (%65.5)^b^	< 0.001*
Roundness Index	25 (%13.6)^a^	18 (%19.8)^a^	80 (%38.8)^b^	< 0.001*
**Laboratory**				
PIV, Median (IQR)	300.7(179.7-426.1)^a^	359.0(240.1-538.4)^b^	499.4(220.9-1005.6)^b^	< 0.001*
PLR, Median (IQR)	108.5(86.8-142.2)^a^	143.1(110.2–192.0)^b^	138.3(85.0-208.8)^b^	< 0.001*

**Fig. 1:**
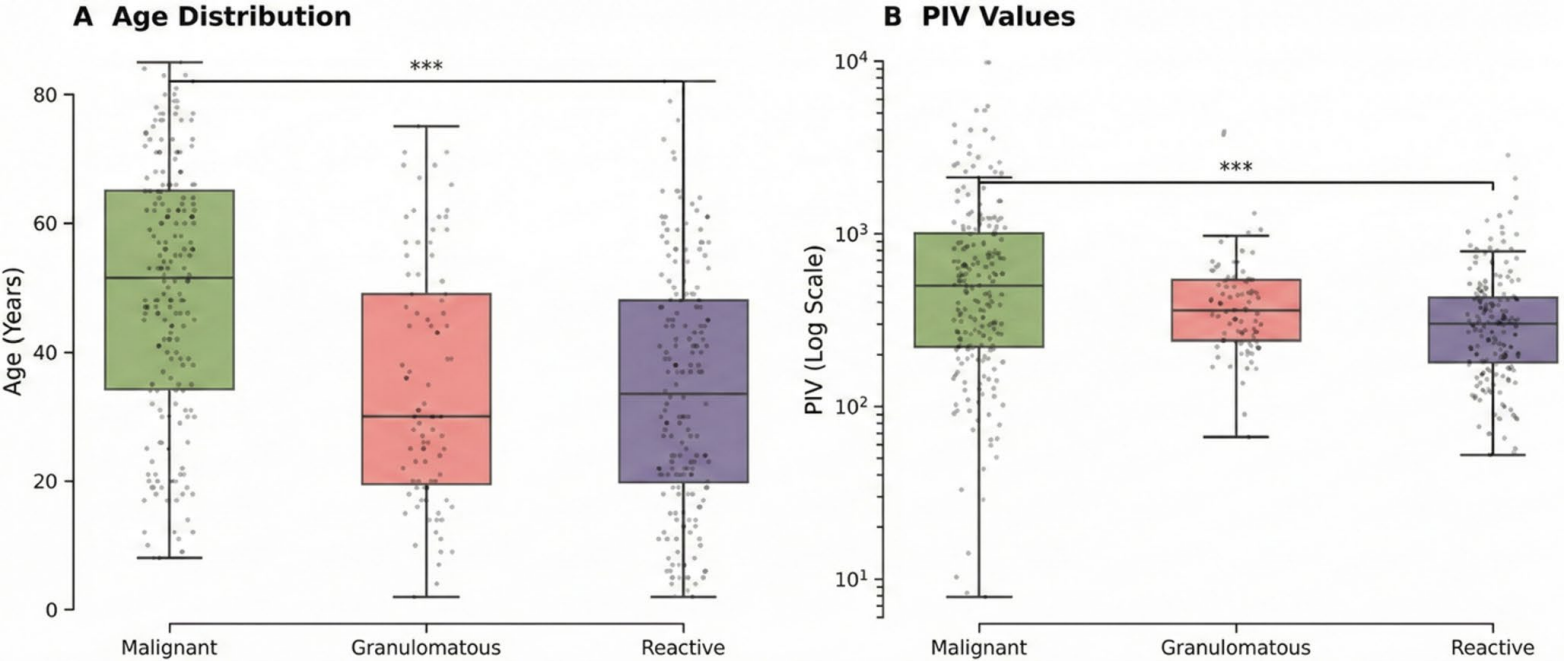
Distribution of malignant and non-malignant (reactive, granulomatous) neck masses according to age and Pan-Immune-Inflammation Value (PIV) levels. **(A)** Distribution of age according to histopathological groups. **(B)** Distribution of PIV levels on a logarithmic scale according to groups. The horizontal lines within the box represent the median, while the points represent individual patient data. (*** = *p* < 0.001, Kruskal-Wallis test)

When inflammatory markers were evaluated, the median Pan-Immune-Inflammation Value (PIV) was found to be statistically significantly higher in the malignant group [499.4 (IQR: 220.9–1005.6)] compared to the reactive [300.7 (IQR: 179.7–426.1)] and granulomatous [359.0 (IQR: 240.0–538.4)] groups (*p* < 0.001) (Fig. 1). Similarly, the median Platelet-Lymphocyte Ratio (PLR) also showed a significant elevation in malignancy (*p* < 0.001).

### Independent risk factors for malignancy prediction

To identify factors predicting malignancy risk, univariate and then multivariate logistic regression analysis was performed (Table 2). Age, gender, ultrasound findings (Loss of fatty hilum, conglomeration, roundness, diameter), laboratory parameters (PIV, PLR), and LAP location, which were found to be significant in the univariate analysis, were included in the multivariate model.

**Table 2:** Results of univariate and multivariate logistic regression analysis of factors predicting malignancy in neck masses. Reference category: Non-malignant group (Reactive and Granulomatous cases). a: Other LAP levels were taken as the reference group

Variables	Univariate Analysis		Multivariate Analysis	
	OR (%95 CI)	*p*	OR (%95 CI)	*p*
**Age**	1.04 (1.03–1.05)	**< 0.001**	1.04 (1.03–1.06)	**< 0.001**
**Gender (Male)**	2.47 (1.66–3.68)	**< 0.001**	3.51 (1.98–6.22)	**< 0.001**
**Conglomeration**	6.12 (4.01–9.36)	**< 0.001**	6.18 (3.54–10.77)	**< 0.001**
**Roundness İndex**	3.40 (2.16–5.34)	**< 0.001**	2.33 (1.25–4.35)	**0.008**
**Hilus Fading**	2.19 (1.46–3.28)	**< 0.001**	1.76 (0.99–3.10)	0.052
**LAP Level** ^**a**^	13.19 (3.47–50.09)	**< 0.001**	7.77 (1.76–34.28)	**0.006**
**Longest Diameter (mm)**	1.03 (1.01–1.04)	0.002	1.02 (0.99–1.04)	0.112
**PIV**	1.001 (1.000–1.002)	**< 0.001**	1.001 (1.000–1.001)	**0.010**
**PLR**	1.003 (1.001–1.005)	0.004	0.99 (0.99–1.00)	0.987

The multivariate logistic regression analysis revealed that the presence of conglomeration (OR: 6.17, 95% CI: 3.54–10.77, *p* < 0.001), male gender (OR: 3.50, 95% CI: 1.97–6.22, *p* < 0.001), Level V placement (OR: 7.77, 95% CI: 1.76–34.28, *p* = 0.006 when Level I-II was taken as reference), and increased roundness index (OR: 2.32, 95% CI: 1.24–4.35, *p* = 0.008) were identified as independent risk factors for malignancy. Additionally, advanced age (OR: 1.04, *p* < 0.001) and high PIV value (OR: 1.001, *p* = 0.010) were other parameters that independently increased the likelihood of malignancy.

Hilus fading (*p* = 0.052), longest diameter (*p* = 0.112), and PLR (*p* = 0.987), which were significant in the univariate analysis, lost their independent predictive qualities in the multivariate model.

### Diagnostic performance and ROC curve analysis

**Fig. 2:**
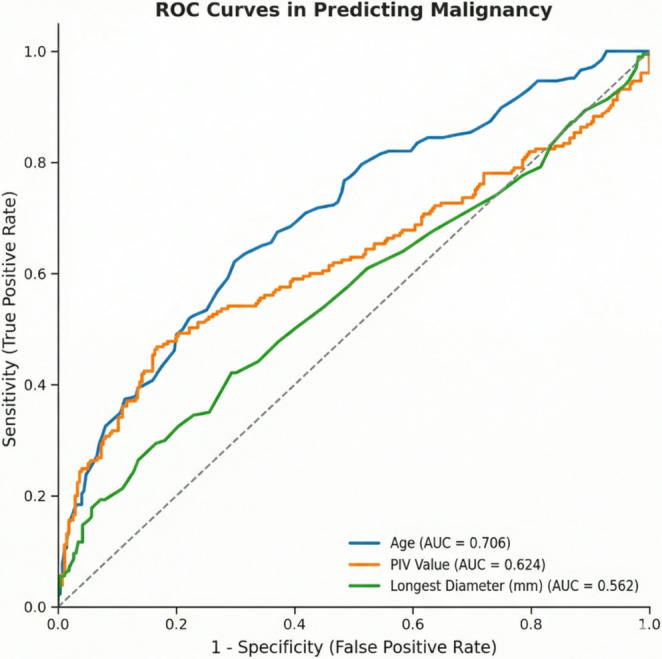
Receiver Operating Characteristic (ROC) curves for Age, PIV, and Longest Diameter parameters in predicting malignancy in patients undergoing neck lymph node dissection. The Area Under the Curve (AUC) was calculated as 0.706 for Age and 0.624 for PIV value. The diagonal dashed line represents the reference line

ROC (Receiver Operating Characteristic) curve analysis was performed to evaluate the predictive accuracy of continuous variables for malignancy (Fig. 2). The area under the curve (AUC) value for age was calculated as 0.706. According to the Youden index, the optimal cut-off value for age was determined to be > 46 years; the sensitivity of being over 46 years of age in detecting malignancy was 62.1%, specificity was 70.2%, positive predictive value (PPV) was 61 and the negative predictive value (NPV) calculated as 71.2%.

The AUC value calculated for PIV among inflammatory markers was 0.624. When a cut-off value of > 567.1 was used for PIV, sensitivity in predicting malignancy was found to be 46.8%, and specificity was high at 83.3% (PPV: 67.6%, NPV: 67.8%). Although the cut-off value calculated for the longest diameter was found to be > 35 mm, its diagnostic value remained lower (AUC: 0.562).

When the diagnostic performance of categorical variables was examined, the PPV of presence of conglomeration on USG for malignancy was found to be 67.4%, and the NPV was 75.0%. In other words, the probability of no malignancy in lymph nodes without conglomeration is 75%. In terms of anatomical localization, the specificity of Level V involvement in predicting malignancy was 88.7% and its PPV was 70.2%.

## Discussion

In this retrospective cohort of 481 patients who underwent cervical lymph node excision, malignancy constituted a substantial proportion of the final diagnoses. The analysis of demographic parameters yielded findings indicating that male gender and increasing age are associated with an elevated risk of malignancy. It has been demonstrated that analogous findings have been observed in a number of studies [11–12].

The subsequent analysis of ultrasonographic variables yielded several notable findings. Conglomeration was determined to be the most robust independent predictor of malignancy (odds ratio [OR] 6.17). The level V localization emerged as a significant predictor (odds ratio [OR] 7.77), followed by an increase in the roundness index (OR 2.32). In clinical practice, loss of fatty hilum, round configuration, and nodal enlargement are often treated as suspicious features; however, our multivariable model suggests that some of these parameters may act as surrogates for more discriminative patterns (such as conglomeration and anatomical level) and may lose independent significance once correlated variables are entered simultaneously.

A notable finding was the high prevalence of conglomeration in malignant cases.

Although the longest diameter differed between groups (*p* = 0.001) and a cut-off of ‘‘35 mm’’ was identified, size alone showed limited discrimination (AUC 0.562) and did not remain an independent predictor in multivariable analysis. This supports the concept that morphology and distribution may be more informative than diameter alone when evaluating cervical LAP.

A significant increase in PIV and PLR values was observed in the malignant group when compared to the non-malignant group using univariate analysis. Furthermore, PIV was found to be independently associated with malignancy in the multivariable model (OR 1.001, *p* = 0.010). This finding suggests that composite inflammation-based indices may reflect tumour-associated systemic inflammation and immune dysregulation in patients presenting with cervical LAP. In the multivariable model employed, PIV was identified as a statistically significant independent predictor of malignancy (OR: 1.001, *p* = 0.010). While an Odds Ratio of 1.001 might appear to be a negligible figure, it is important to note that it represents the incremental risk associated with a single-unit increase in the PIV score. In view of the wide range of PIV values observed in the study population, the cumulative effect of these increments is of clinical relevance. However, for bedside clinical application, the use of a continuous scale is less practical than a categorical threshold. Consequently, a cut-off value of 570 was determined through ROC analysis, yielding a high specificity of 83.3%. This dichotomous approach (PIV > 570) permitted the integration of PIV into a simplified clinical scoring system, thereby transforming a complex inflammatory marker into an actionable diagnostic tool. From a diagnostic-performance standpoint, PIV provided modest overall discrimination (AUC 0.624) but high specificity at the selected cut-off (567.1; specificity %83,3).

Level V involvement was independently associated with malignancy and showed high specificity (%88,7). This finding is in line with prior reports suggesting that Level V nodal disease is more frequently associated with malignant processes [11].

Taken together, our findings support a pragmatic, preoperative risk stratification based on (i).

demographic risk (age > 46 years), (ii) high-risk ultrasonographic patterns (conglomeration and increased roundness index), (iii) high-risk localization (Level V), and (iv) a high-specificity laboratory adjunct (PIV > 567.1). In a real-world workflow, combining these dimensions may help prioritize patients for biopsy, guide the urgency of referral, and improve counseling about the likelihood of malignancy.

The study’s key strengths include the large sample size, the utilisation of excisional histopathology as the reference standard, and the simultaneous evaluation of demographic variables, neck level, ultrasonographic morphology, and inflammation-based indices (PIV/PLR). Furthermore, the utilisation of both broad diagnostic categories (reactive/granulomatous/malignant) and specific diagnoses has been shown to enhance clinical interpretability.

The present study is subject to limitations inherent to its retrospective design, including selection bias (given that only excised nodes were included) and potential residual confounding from comorbid inflammatory or infectious conditions that may influence blood indices. Ultrasonographic assessment is operator and technique-dependent; interobserver variability and the lack of standardized reporting may have affected the measured prevalence of morphological features. Finally, cut-off values derived from ROC analysis may not generalize across institutions and should be externally validated.

Prospective multicenter studies should validate the observed associations and evaluate whether a combined clinical–radiological–laboratory score improves decision-making beyond standard imaging assessment. Further work should also clarify the incremental value of PIV over more established inflammation indices and determine clinically actionable thresholds that balance missed malignancies against unnecessary excisions.

### Proposed risk stratification and clinical scoring system

To translate our statistical findings into clinical practice, we developed a preliminary risk stratification score based on the independent predictors identified in our multivariate analysis (Table 3). The score assigns points to six key variables: conglomeration (+ 3), age > 46 years (+ 2), PIV > 570 (+ 2), level V location (+ 2), male sex (+ 1) and increased roundness index (+ 1).

**Table 3:** Proposed malignancy risk scoring system for cervical lymphadenopathy

A. Scoring System		Points
Conglomeration		+3
Age >46 years		+2
PIV >570		+2
Level V location		+2
Male Sex		+1
Roundness index		+1
Total possible score		0-11
B. Risk Stratification	Score Range	Recommended Clinical Approach
Low risk	0-1	Observation / Medical Therapy Prioritize follow-up. Consider granulomatous diseases in female patients.
Moderate risk	2-5	Diagnostic Biopsy (Gray Zone) Malignancy cannot be ruled out. Tissue diagnosis (FNAB or Core Biopsy) is required.
High risk	6-11	Excisional Biopsy Strong suspicion of malignancy. Prompt excision or oncological consultation is advised.

Based on our cohort, patients were stratified into three risk groups:

- Low risk (0–1 points): In our study, the malignancy rate in this group was only 7.4%. For these patients, especially in the absence of red flags, a ‘‘wait-and-scan’’ approach with antibiotic therapy may be prioritized to avoid unnecessary invasive procedures. However, granulomatous diseases (e.g., tuberculosis) should be considered in female patients within this group.
- Moderate risk (2–5 points): This ‘’gray zone’’ carried a malignancy rate of 36.2%. We strongly advocate for tissue diagnosis via Fine Needle Aspiration Biopsy (FNAB) or core biopsy for this group before proceeding to excision.
- High risk (6–11 points): With a malignancy rate of 83.1% (reaching 100% at higher scores), these patients require prompt excisional biopsy or direct oncological consultation.

## **Conclusion**

This study demonstrates that combining demographic, ultrasonographic, and laboratory parameters offers a clinically significant and feasible approach to predicting malignancy risk in patients undergoing excisional biopsy for cervical lymphadenopathy. Our findings revealed that factors such as male gender, advanced age, Level V lymph node location, presence of conglomeration, increased roundness index, and high Pan-Immune-Inflammation Value (PIV) were independently associated with malignancy. Although this scoring system demonstrated high discriminatory performance in our study population, external validation in larger, multicenter cohorts is needed to confirm its universal applicability.

## Data Availability

All the data generated or analyzed during this study are included in this published article.
